# Peroxiporins Are Induced upon Oxidative Stress Insult and Are Associated with Oxidative Stress Resistance in Colon Cancer Cell Lines

**DOI:** 10.3390/antiox10111856

**Published:** 2021-11-22

**Authors:** Ana Čipak Gašparović, Lidija Milković, Claudia Rodrigues, Monika Mlinarić, Graça Soveral

**Affiliations:** 1 Ruđer Bošković Institute, Bijenička 54, HR-10000 Zagreb, Croatia; Lidija.Milkovic@irb.hr (L.M.); Monika.Mlinaric@irb.hr (M.M.); 2 Research Institute for Medicines (iMed.ULisboa), Faculty of Pharmacy, Universidade de Lisboa, 1649-003 Lisboa, Portugal; claudiarf@medicina.ulisboa.pt

**Keywords:** aquaporin, colon cancer cell line, oxidative stress, NRF2

## Abstract

Oxidative stress can induce genetic instability and change cellular processes, resulting in colorectal cancer. Additionally, adaptation of oxidative defense causes therapy resistance, a major obstacle in successful cancer treatment. Peroxiporins are aquaporin membrane channels that facilitate H_2_O_2_ membrane permeation, crucial for regulating cell proliferation and antioxidative defense. Here, we investigated four colon cancer cell lines (Caco-2, HT-29, SW620, and HCT 116) for their sensitivity to H_2_O_2_, cellular antioxidative status, and ROS intracellular accumulation after H_2_O_2_ treatment. The expression of peroxiporins AQP1, AQP3, and AQP5 and levels of NRF2, the antioxidant transcription factor, and PPARγ, a transcription factor that regulates lipid metabolism, were evaluated before and after oxidative insult. Of the four tested cell lines, HT-29 was the most resistant and showed the highest expression of all tested peroxiporins and the lowest levels of intracellular ROS, without differences in GSH levels, catalase activity, nor NF2 and PPARγ levels. Caco-2 shows high expression of AQP3 and similar resistance as HT-29. These results imply that oxidative stress resistance can be obtained by several mechanisms other than the antioxidant defense system. Regulation of intracellular ROS through modulation of peroxiporin expression may represent an additional strategy to target the therapy resistance of cancer cells.

## 1. Introduction

The analysis of colorectal cancer (CRC) cases in 40 European countries in 2018 revealed CRC as the second most common cancer diagnosed for women and the third for men [1]. The genes known to be involved in CRC development include *APC*, *RAS*, *BRAF*, *ERBB2* (*HER2*), as well as MMR (mismatch repair) status [2]. Some of these genes are used to monitor patients’ status and to define a strategy for cancer treatment [2]. Further, genetic instability, as one of the hallmarks of cancer, introduces additional mutations, thereby destabilizing normal transcription patterns and transcription regulation. These events support tumor growth, progression, and therapy resistance [3]. Additionally, inflammation and oxidative stress can also introduce new mutations, further supporting tumor growth and therapy resistance [4]. Oxidative stress, an imbalance of the cellular redox status toward oxidation, increases reactive oxygen species (ROS), which then regulate cellular processes. When summed, these events boost cancer growth and therapy resistance. To counteract some of the negative consequences of oxidative stress, cells increase their antioxidative defense, especially via the nuclear factor erythroid 2 [NF-E2]-related factor 2/Kelch-like ECH-associated protein 1 (NRF2/KEAP1) signaling pathway [5,6]. However, while activation of this pathway is protective for the cell and is a desirable event for normal cells, in cancer cells, it is unfavorable and leads to adaptation to stress and therapy resistance [6]. As some conventional chemotherapies are based on or additionally increase cellular oxidative stress, such as doxorubicin, cisplatin, 5-fluorouracil, or oxaliplatin [7,8], the study of strategies to control ROS production or fluxes is paramount.

Cancer growth is supported by changes in the cell’s metabolic pathways, which are caused either by the abovementioned genetic instability or by changes in transcription factors. Peroxisome proliferator-activated receptors (PPAR) are a group of ligand-activated transcription factors that regulate lipid metabolism, immune response, and glucose metabolism [9]. PPARγ is part of the PPAR family, mostly present in adipose tissue [10]. Dysregulation of PPARγ in cancer is observed, with evidence supporting both its anti-tumorous and pro-tumorigenic effects [9,11].

Aquaporins (AQPs) are transmembrane proteins mainly responsible for the transport of water, glycerol, small molecules (urea, hydrogen peroxide), gases, and ions [12,13]. These proteins are important for cell homeostasis and are widely expressed in humans, showing specific organ, tissue, and cellular localization [14], thus suggesting a relation between their expression and function in each organ. The 13 AQP isoforms identified so far are grouped into three families according to their selectivity and primary sequence: (i) classical or orthodox aquaporins, which are considered selective for water (AQP1, AQP2, AQP4, AQP5, AQP6, and AQP8); (ii) aquaglyceroporins, which, in addition to water, facilitate the transport of small uncharged solutes, such as glycerol and urea (AQP3, AQP7, AQP9, and AQP10); and (iii) S-aquaporins with lower sequence homology, unique subcellular localization, and with still unclear selectivity of their permeability (AQP11 and AQP12) [15,16]. AQPs are involved in numerous physiological mechanisms related to water and glycerol transport, including renal water absorption, exocrine fluid secretion, epidermal hydration, neuro-homeostasis, and fat metabolism. In tumors, recent studies showed an abnormal AQP expression level and the involvement of AQPs in processes like regulation of cell volume and shape, energy metabolism, cell migration, adhesion, proliferation, and differentiation [17,18,19]. Concomitantly, their dysregulation contributes to tumor-associated edema, tumor-cell proliferation and migration, as well as tumor angiogenesis and metastasis [19]. More recently, a group of aquaporins was found to facilitate the diffusion of H_2_O_2_ through lipid membranes and has been identified as peroxiporins (AQP1, AQP3, AQP5, AQP8, AQP9, and AQP11) [20,21,22]. Several studies point to a functional relationship between peroxiporins and tumor progression and proliferation [23].

In recent years, AQPs have been highlighted as target molecules in the search for good-quality prognostic markers, as well as molecular targets for tumor therapeutics [24]. In particular, AQP1, AQP3, and AQP5 were found to be correlated with lymph node metastases in colon cancer patients [25]. Additionally, the expression of these aquaporins was increased in cancer tissues [26,27], and the inhibition of AQP3 permeability reduced tumor progression in a murine colon cancer model [28]. Taken that these three aquaporins channel H_2_O_2_, our aim was to investigate whether they support tumor growth through regulation of cellular ROS and antioxidative defense.

## 2. Materials and Methods

### 2.1. Cell Lines

The four colon cancer cell lines, Caco-2 (ATCC, HTB-37), HT-29 (ATCC, HTB-38), SW620 (ATCC, CCL-227), and HCT 116 (ATCC, CCL-247), were cultivated in Dulbecco’s Modified Eagle’s Medium (DMEM; SigmaAldrich, St. Louis, MO, USA) supplemented with 10% fetal calf serum (FCS, SigmaAldrich, St. Louis, MO, USA) in a humidified incubator with 5% CO_2_ maintained at 37 °C. The medium was changed every 2–3 days, and experiments were performed with 70% to 80% cell confluence.

### 2.2. Cell Viability

All four cell lines were trypsinized, counted, and were then seeded in a 96-well plate (TPP, Trasadingen, Switzerland) at a density of 1 × 10^4^ cells per well and were allowed to adhere for 24 h. Cells were then treated with a range of H_2_O_2_ concentrations up to 250 µM for 24 h. Cell viability was determined by MTT EZ4U assay (Biomedica, Vienna, Austria) according to the manufacturer’s instructions. Briefly, treated cells were incubated with 20 µL of colorless solution for 2 h. During this time, the solution was oxidized in the mitochondria of living cells and formed a yellow product, the color intensity of which was measured with a Multiskan EX plate reader (Thermo Electron Corporation, Shanghai, China) at 450 nm, with 620 nm as reference.

### 2.3. Glutathione (GSH) Levels and Catalase Activity

For the GSH measurement, all four cell lines were trypsinized, counted, and seeded in a 6-well plate (TPP, Trasadingen, Switzerland) at 0.5 × 10^6^ cells per well and were allowed to adhere for 24 h. Next, cells were treated with 100 µM H_2_O_2_ for 24 h, trypsinized, centrifuged, and stored as dry pellet at –80 °C. The intracellular GSH content was measured by a modification of the protocol described by Tietze [29]. Briefly, the protein concentration of samples was measured by the Bradford method [30] and then diluted to 0.03 mg/mL protein for normalization. For the GSH assay, 150 μL of sample or GSH standards was pipetted per well, and then reaction mix (1.8 mM 5,5-dithio-bis-2-nitrobenzoic acid, 0.4 U GSH reductase, and 0.6 mM NADPH in phosphate buffer: 100 mM NaH_2_PO_4_, 5 mM EDTA pH 7.4) was added to start the reaction. Color formation was measured spectrophotometrically in a Multiskan EX plate reader (Thermo Electron Corporation, Shanghai, China) at 405 nm. GSH concentration in cell lysates was expressed as µM of GSH per milligram of total protein (nmol/mg). Catalase activity was measured by a modification of the protocol described by Goth [31]. Briefly, 40 µL of samples was mixed with 65 mM hydrogen peroxide and was left for 5 min to allow the catalase to degrade the H_2_O_2_. Ammonium molybdate (32.4 mM) was used as a stop solution to chelate the remaining H_2_O_2_, creating a yellow complex. The colour development was measured spectrophotometrically in a Multiskan EX plate reader (Thermo Electron Corporation, Shanghai, China) at 405 nm. Catalase activity is expressed as units per milligram of proteins in cell lysate (U mg^−1^), where one unit of catalase activity is defined as the amount of enzyme needed for degradation of 1 μmol of H_2_O_2_/min at 25 °C.

### 2.4. ROS

To measure ROS levels, all four cell lines were seeded in a black 96-well plate (Thermo Fisher Scientific, Nunc A/S, Roskilde, Denmark) at 1 × 10^4^ cells per well and were allowed to adhere for 24 h. Cells were then incubated with 10 µM non-fluorescent probe 2′,7′-dichlorodihydrofluorescein diacetate (DCFH-DA; Sigma-Aldrich, St. Louis, MO, USA) for 1 h at 37 °C in 5% CO_2_ to allow the dye to penetrate the cells. After the excess of dye was removed, cells were washed once with PBS and treated with 250 µM H_2_O_2_. The fluorescence was measured before H_2_O_2_ treatment and 90 min after treatment with a Tecan Infinite M200 spectrofluorometer (Tecan, Männedorf, Switzerland), with excitation at 500 nm and emission detection at 530 nm.

### 2.5. RT-PCR

For determination of gene expression by RT-PCR, cells were trypsinized, counted, and plated in a 6-well plate at a density of 0.5 × 10^6^ cells per well. The next day, cells were treated with 100 µM H_2_O_2_ or with media as a control for another 24 h, at which point they were harvested in TRI reagent (Ambion, Austin, TX, USA). Total RNA was isolated according to the manufacturer’s instructions, and contaminating DNA was removed using RNase-free DNase I (Sigma Aldrich, St. Louis, MO, USA). The RNA concentration was determined using a NanoDrop-1000 spectrophotometer (NanoDrop Technologies, Wilmington, DE, USA). To obtain cDNA, 1 µg of total RNA was used for the reverse transcription reaction with oligo dT23 primer (Sigma Aldrich, St. Louis, MO, USA) using MultiScribe reverse transcriptase (Applied Biosystems, Thermo Fisher Scientific, Waltham, MA, USA).

Real-time PCR reactions were carried out using a CFX96 Real-Time System C1000 (BioRad, Hercules, CA, USA), the TaqMan Universal PCR Master Mix (Applied Biosystems, Thermo Fisher Scientific, Waltham, MA, USA), and the following specific TaqMan pre-designed gene expression primers: AQP1 (Hs01028916_m1), AQP3 (Hs01105469_g1), and AQP5 (Hs00387048_m1) (Applied Biosystems, Thermo Fisher Scientific, Waltham, MA, USA). The relative quantification of gene expression was determined using the 2^−ΔCt^ method (adapted from [32]), with β-actin as endogenous control. All samples were run in triplicate, and the average values were calculated.

### 2.6. Western Blot

Cells cultivated to 80% confluency were trypsinized, counted with Trypan blue exclusion method, plated in 6-well plates (TPP, Trasadingen, Switzerland) at a density of 0.5 × 10^6^ cells per well, and left for 24 h to attach. The next day, cells were treated with 100 µM H_2_O_2_ and left for an additional 24 h. All four cell lines were used for this experiment.

Cells were lysed with RIPA buffer (20 mM Tris-HCl pH 7.5, 150 mM NaCl, 1% Triton X, 0.5% sodium deoxycholate, 0.1% sodium dodecyl sulfate (SDS)) containing protease inhibitors (Roche Diagnostics GmbH, Mannheim, Germany), and supernatants were collected for Western blot analyses. Protein concentration was measured with the Bradford method [30]. Protein samples were mixed with Laemmli buffer and boiled for 5 min at 95 °C. At total of 20 µg of total proteins was resolved on the Tris-glycine SDS-PAGE gels (9%), transferred to nitrocellulose membranes (Roti^®^-NC, Carl Roth, Karlsruhe, Germany), and stained with Ponceau S solution (Sigma Aldrich, St. Louis, MO, USA) for evaluation of transfer efficacy. Membranes were further blocked with 5% nonfat milk (Cell Signaling Technology (CST), Danvers, MA, USA) in Tris-buffered saline (TBS; 50 mM Tris-Cl, 150 mM NaCl, pH 7.6) containing 0.1% Tween-20 for one hour, and incubated with primary antibodies overnight at 4 °C. The primary antibodies used were: rabbit monoclonal antibodies for NRF2 (CST:#12721), PPARγ (CST:#2435), and β-actin (CST:#8457). After incubation with horseradish peroxidase-conjugated secondary anti-rabbit IgG (CST:#7074), immunoreactive bands were visualized using the SuperSignal^TM^West Pico PLUS Chemiluminescent Substrate (Thermo Scientific, Rockford, IL, USA) and Alliance Q9 Mini (UVITEC, Cambridge, UK). The imaging software Nine Alliance was used for the analysis and quantification of levels of protein expression. Normalization was made with total proteins (Ponceau S staining) and with β-actin as a loading control.

### 2.7. Statistical Analyses

All experiments were performed in biological and technical triplicates. Mean values were compared using two-way ANOVA with Tukey’s post hoc test or Student’s *t*-test, using Prism GraphPad 8.0 (GraphPad Software, San Diego, CA, USA). Values of *p* < 0.05 were considered significant.

## 3. Results

### 3.1. Cell Viability

**Figure 1:**
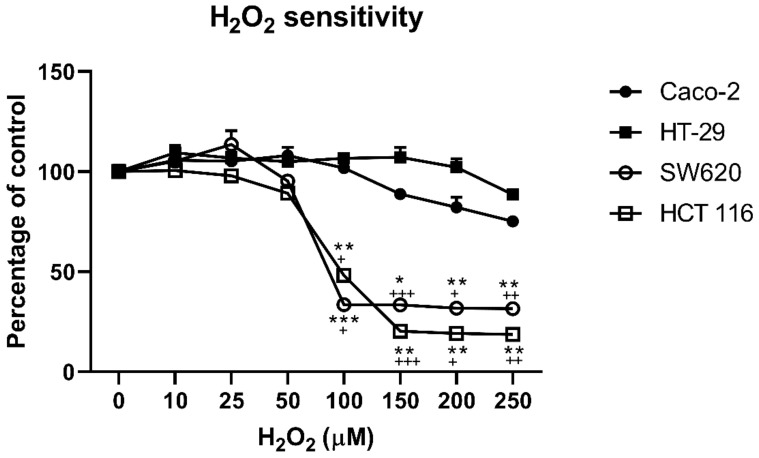
Viability of SW620, Caco-2, HT-29, and HCT 116 cell lines after H_2_O_2_ challenge. All four cell lines were treated with a range of H_2_O_2_ concentrations for 24 h, at which point their viability was measured by MTT assay. The results present the mean ± SD from three independent experiments. Significance levels: * and + *p* < 0.05; ** and ++ *p* < 0.01; *** and +++ *p* < 0.001. + difference between the indicated cells lines (SW620 or HCT 116, respectively) and Caco-2 cell line; * difference between the indicated cells line (SW620 or HCT 116, respectively) and HT-29 cell line.

To study the role of aquaporins in colon cancer cell lines, first, we tested the sensitivity of four colon cancer cell lines to hydrogen peroxide (Figure 1). Interestingly, the MTT assay showed that at lower H_2_O_2_ concentration, up to 50 µM, there were no differences between the four cell lines. At 100 µM to 250 µM, H_2_O_2_ SW620 and HCT 116 showed a statistically significant drop in cell viability (*p* < 0.001), while Caco-2 showed a slight but significant decrease in viability compared to the control (*p* = 0.013).

### 3.2. GSH Levels and Catalase Activity

**Figure 2:**
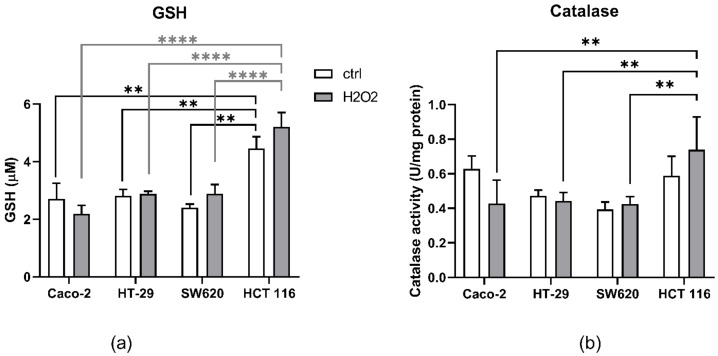
Antioxidant status of Caco-2, HT-29, SW620, and HCT 116 after 100 µM H_2_O_2_ challenge: (**a**) total GSH levels before and after H_2_O_2_ challenge in all four cell lines; (**b**) catalase activity before and after H_2_O_2_ challenge in all four cell lines. The results present the mean ± SD from three independent experiments. Significance levels: ** *p* < 0.01 and **** *p* < 0.0001.

Next, we evaluated the antioxidative defense system by measuring the levels of GSH, as well as catalase activity, before and after treatment with H_2_O_2_ (Figure 2). Interestingly, the HCT 116 line had the highest GSH levels, regardless of H_2_O_2_ treatment. Additionally, 100 µM H_2_O_2_ did not reduce the GSH levels in either of the used cell lines. Catalase activity was similar in all untreated cell lines. While H_2_O_2_ treatment did not change catalase activity in comparison to untreated cells, a significant difference was observed between the HCT 116 cell line treated with H_2_O_2_ in comparison to other cell lines (Caco-2, HT-29, and SW620) treated with H_2_O_2._

### 3.3. Intracellular ROS

**Figure 3:**
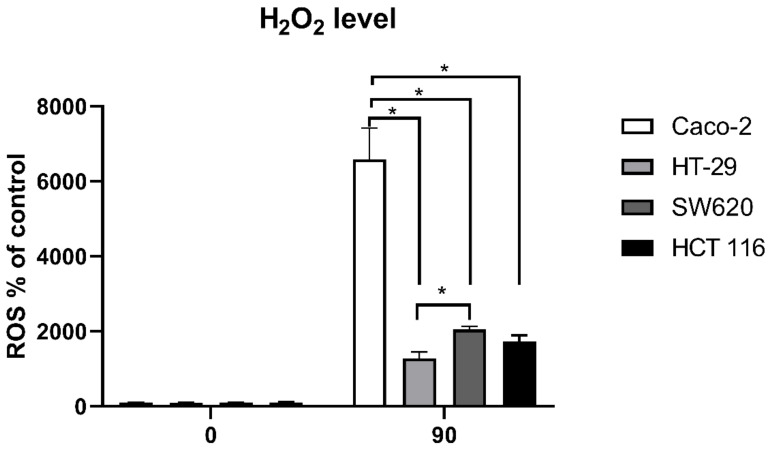
Intracellular ROS levels of Caco-2, HT-29, SW620, and HCT 116 cell lines. The fluorescence due to ROS accumulation triggered by 250 µM H_2_O_2_ treatment was followed over 90 min. The results present the mean ± SD from three independent experiments. Significance levels: * *p* < 0.05.

Next, we measured the intracellular ROS accumulation in the four cell lines (Figure 3). Following the oxidative treatment with H_2_O_2_, Caco-2 had the highest levels of intracellular ROS (*p* < 0.05), whereas the HT-29 cell line had significantly lower levels of intracellular ROS compared to Caco-2 and SW620 (*p* < 0.05).

### 3.4. AQP1, AQP3, and AQP5 Expression

**Figure 4:**
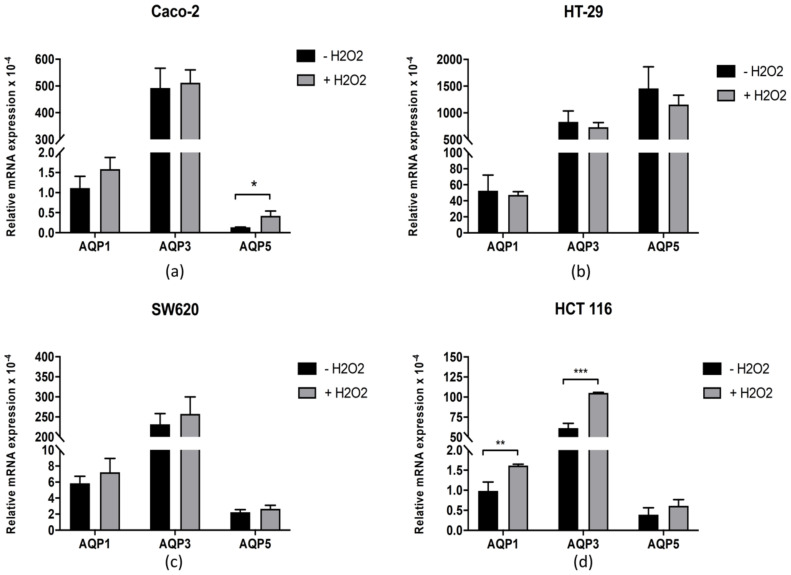
Effect of oxidative stress on AQP1, AQP3, and AQP5 expression. AQP1, AQP3, and AQP5 mRNA expression level in (**a**) Caco-2, (**b**) HT-29, (**c**) SW620, and (**d**) HCT 116 cell lines. Cells were incubated (or not) with 100 µM H_2_O_2_ for 24 h at 37 °C in 5% CO_2_. Data represent mean ± SD from three independent experiments. Significance levels: * *p* < 0.05, ** *p* < 0.01 and *** *p* < 0.001.

As peroxiporins permeate H_2_O_2_ across membranes, we measured the expression of AQP1, AQP3, and AQP5 in all four cell lines before and after treatment with H_2_O_2_ (Figure 4). The results show that regardless of the oxidative treatment, in HT-29 and SW620 cell lines, the levels of all three aquaporins did not change significantly. In the Caco-2 cell line, no differences were found for AQP1 and AQP3 expression levels after treatment, but the very low initial level of AQP5 increased significantly. Unlike Caco-2, HCT 116 did not show changes in AQP5, but H_2_O_2_ treatment significantly increased the levels of AQP1 and AQP3.

### 3.5. Western Blot

**Figure 5:**
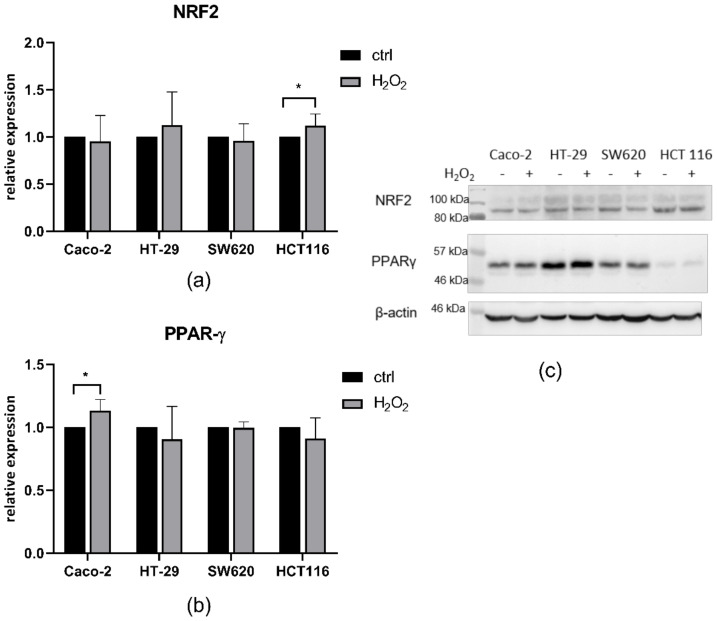
Western blot analysis of (**a**) NRF2 and (**b**) PPARγ in Caco-2, HT-29, SW620, and HCT 116 before and after 100 µM H_2_O_2_ treatment; (**c**) representative photo of the membrane with molecular weights of protein standards. Data represent mean ± SD from three independent experiments.* *p* < 0.05.

We measured the protein levels of the two transcription factors, NRF2 and PPARγ (Figure 5). Interestingly, neither NRF2 nor PPARγ changed significantly in HT-29 nor SW620 cell lines after H_2_O_2_ treatment. In the HCT 116 cell line, NRF2 increased significantly, while PPARγ did not change after H_2_O_2_ treatment; whereas, in the Caco-2 cell line, NRF2 did not change due to treatment, while PPARγ increased significantly after treatment.

## 4. Discussion

In this study, we report that resistance to oxidative stress is not necessarily related to an increased antioxidative system, but regulation of H_2_O_2_ permeation can also contribute to the resistant phenotype of the tumor. We hypothesized that aquaporins with peroxiporin activity could modify the cellular antioxidative defense system, thereby contributing to oxidative stress resistance.

Oxidative stress is the result of interactions between different risk factors in CRC, further enhancing pathways of cancer development [33]. Oxidative stress contributes to cancer development either through metabolic changes, which support ROS formation, or by being a risk factor that initially damages cells, resulting in transformation [34]. Both these events result in higher ROS content that overwhelms the cell’s ability to neutralize the excess of ROS, which, in turn, damages DNA, further supporting mutation, genetic instability, and changes in signaling pathways, creating a vicious circle [35]. The ability to control the intracellular levels of ROS, not only by neutralization but also by controlling ROS fluxes in both directions through the cell membrane, is a strategy to survive oxidative stress. Therefore, peroxiporins are the best candidates for H_2_O_2_ flux control. An association between aquaporins and cancer was found for a variety of cancers, including colorectal cancer [36], which is supported by the role of aquaporins in cellular migration, proliferation, and adhesion [18,37].

To ascertain the role of aquaporins in the modulation of signaling pathways in colon cancer, we used four colon cancer cell lines—Caco-2, HT-29, SW620, and HCT 116—with different sensitivities to oxidative stress, mediated by H_2_O_2_. HT-29 and Caco-2 cell lines were more resistant to oxidative stress, in contrast to SW620 and HCT 116. Differences in H_2_O_2_ sensitivity can arise via different mechanisms: either from a high antioxidative defense system or by a controlled influx of the stressor (H_2_O_2_). To determine whether these differences are due to the antioxidant system, we tested glutathione levels and catalase activity before and after H_2_O_2_ treatment. Catalase is the enzyme with the highest turnover number [38], and is thereby the first to neutralize H_2_O_2_. If H_2_O_2_ is not neutralized by catalase, the GSH system readily reacts with this oxidant, neutralizing it either by direct interaction or through GSH enzymatic cascade [39]. Surprisingly, GSH levels were the highest in the most sensitive cell line—HCT 116 cells, both control and H_2_O_2_ treated. Moreover, catalase activity increased significantly after H_2_O_2_ treatment only in the HCT 116 cell line, indicating a certain level of antioxidant system activation. These results indicate that other factors among antioxidant defense can also dictate sensitivity to H_2_O_2_.

The next step was to follow whether this difference is the result of lower intracellular levels of ROS induced by H_2_O_2_ treatment. Our results confirm that for the HT-29 cell line, the level of ROS was indeed the lowest and did not reach the level of other cell lines after 90 min. Yet, the Caco-2 cell line, which manifested the same level of H_2_O_2_ sensitivity as HT-29, had the highest ROS level, in contrast to SW620 and HCT 116, which had ROS levels similar to HT-29 but both of which were more sensitive to the H_2_O_2_ challenge. Since H_2_O_2_ is channelled through aquaporins with peroxiporin activity, we measured the expression of AQP1, AQP3, and AQP5. Interestingly, HT-29 had the highest relative expression of all three measured peroxiporins. Further, the Caco-2 cell line, with oxidative stress resistance similar to that of HT-29, had increased AQP5 after H_2_O_2_ treatment and, similarly to HT-29, a high relative expression of AQP3. Recent studies indicate that in lung adenocarcinoma, higher AQP3 transcript levels in cancer tissues were related to poor prognosis, and silencing of AQP3 decreased H_2_O_2_-induced proliferation [40]. In addition, high expression of AQP3 in triple-negative breast cancer was associated with a worse prognosis, while in HER2-positive breast cancer, AQP3 was in the group of genes predicting worse relapse-free survival after neoadjuvant chemotherapy plus either trastuzumab and/or lapatinib [41]. The exact mechanism by which AQP3 achieves its effect on tumor resistance is not clear, but it could be through regulation of ROS levels, as SW620 and HCT 116 had a lower relative expression of AQP3 than the two resistant cell lines. Another possibility is AQP3 acting in combination with other aquaporins, such as AQP1 and AQP5. In our study, SW620 and HCT 116 had a lower relative expression of AQP1 and AQP5 compared to HT-29 and a lower expression of AQP3 compared to both Caco-2 and HT-29 cell lines. These results are in line with previously published data showing that inhibition of AQP1 and AQP5 in HT-29 cells with high expression of these two aquaporins reduced their ability to migrate, while this inhibition did not affect HCT 116 cells with low expression of these aquaporins [42]. The authors conclude that HCT 116 and HT-29 have an alternative mechanism to migrate. Similarly, the four cell lines have different mechanisms to cope with the oxidative challenge, and resistance can be achieved by high aquaporin expression. It should be noted that aquaporins channel H_2_O_2_ within minutes after exposure and after the initial rapid increase the plateau is established [22], thereby enabling the cells to cope with the exogenous H_2_O_2_, though not necessarily by antioxidant defense upregulation, as this did not lead to resistance in HCT 116 cells. Finally, to evaluate whether oxidative challenge includes activation of specific signalling pathways, the levels of NRF2 and PPARγ transcription factors were evaluated before and after H_2_O_2_ treatment. Interestingly, NRF2 significantly increased after H_2_O_2_ treatment only in HCT 116 cells. Yet, this increase in NRF2 levels, although significant, is very small and therefore cannot be considered physiologically relevant. These results show that the antioxidant transcription factor NRF2 does not necessarily have to be involved in the resistance to oxidative challenge. Unexpectedly, PPARγ was significantly increased after H_2_O_2_ treatment only in Caco-2 cells and correlated with increased AQP5 expression. PPARγ has an important role in the regulation of adipose tissue and lipid metabolism [43], and its inhibition is also correlated with the downregulation of AQP3 in keratinocytes [44]. Moreover, AQP5 depletion impaired adipocyte differentiation in an adipose cell line, reducing lipid droplets and affecting the adipocyte morphology together with downregulation of differentiation markers [45]. Therefore, it is not surprising that PPARγ correlates with AQP5 expression, although a correlation with AQP3 that channels glycerol and may contribute to lipid trafficking could also be expected.

## 5. Conclusions

Altogether, our results indicate that stress resistance can be induced by several different and unrelated mechanisms that do not necessarily include antioxidative defense factors. Modulation of intracellular ROS levels by specific aquaporins deserves further investigation as a novel strategy for cancer therapeutics.

## Data Availability

Data is contained within the article.
